# A Rare Localization of Ectopic Pregnancy: Intramyometrial Pregnancy in Twin Pregnancy following IVF

**DOI:** 10.1155/2014/893935

**Published:** 2014-03-18

**Authors:** Lahcen Boukhanni, Yassir Ait Benkaddour, Ahlam Bassir, Abdrahim Aboulfalah, Hamid Asmouki, Abderraouf Soummani

**Affiliations:** ^1^Department of Obstetrics and Gynecology, University Hospital of Marrakesh, Cadi Ayyad University, 40 000 Marrakesh, Morocco; ^2^Department of Obstetrics & Gynecology, Mother and Child Hospital, Mohammed VI University Hospital, Cadi Ayyad University, School of Medicine, 40 000 Marrakesh, Morocco

## Abstract

Intramyometrial pregnancy is a rare form of ectopic pregnancy. It makes a diagnostic and therapeutic challenge. If misdiagnosed the intramyometrial pregnancy can cause a uterine rupture and become life-threatening condition. We report a case of intramyometrial pregnancy in twin pregnancy following IVF with spontaneous abortion of the first twin At 9 weeks of gestation. The 10 weeks scan showed a normal fetus which was described to be highly localized in the uterus but the diagnosis of intramyometrial pregnancy was not suspected. The patient was admitted at 14 weeks of gestation with pelvic pain, hemorrhage, and shock. She was operated and the diagnosis of ruptured intramyometrial pregnancy was done and managed conservatively. This case illustrates the diagnostic difficulties of intramyometrial pregnancy. We discuss pathophysiology, diagnosis, and treatment of this exceptional form of ectopic pregnancy.

## 1. Introduction

Intramyometrial pregnancy is the rarest subtype of ectopic pregnancy. The first case of intramural pregnancy was reported in 1924. Preoperative diagnosis is difficult and hysterectomy is always required because of delayed diagnosis.

## 2. Case Report

**Figure 1:**
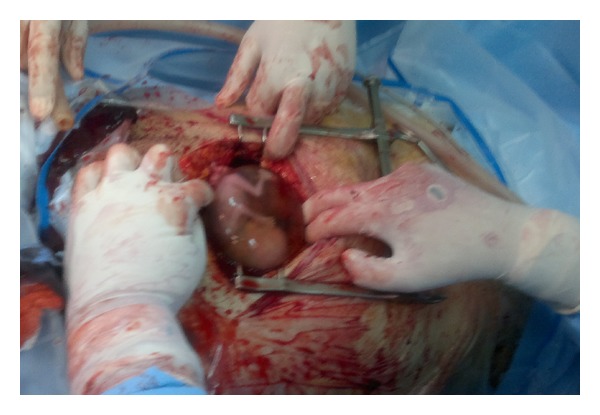
Partial rupture of the right lateral uterine.

**Figure 2:**
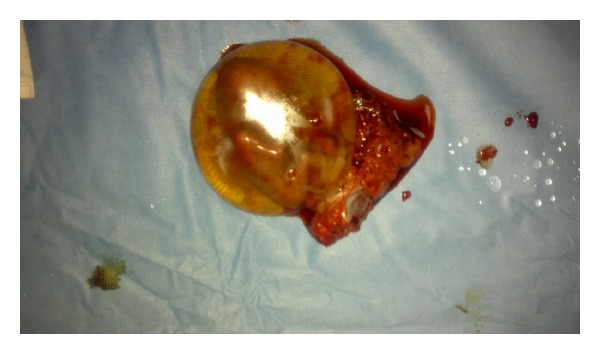
Conceptus adherent to myometrium.

We report a case of intramyometrial pregnancy in twin pregnancy following IVF. The 32-year-old patient had a history of 4 years of unexplained infertility with 3 implantation failures following IVF. She became pregnant of a twin after an IFV-ET of 2 embryos in our department. The embryo transfer was easy and without touching the uterine wall. A spontaneous abortion of the first twin occurred at 9 weeks of gestation. The 10 weeks scan showed a normal fetus which was described to be highly localized in the uterus but the diagnosis of intramyometrial pregnancy was not suspected. The patient was admitted at 14 weeks of gestation with pelvic pain, hemorrhage, and shock. Ultrasound showed an exocentric gestational sac with cardiac activity and suspected rupture of intramyometrial pregnancy. The patient was immediately operated. Surgical exploration showed a partial rupture of the right lateral uterine wall with a conceptus adherent to myometrium without communication with the uterine cavity (Figures 1 and 2). The conceptus was removed and the uterine wall closed with a 2 layers of resorbable sutures. Followup was free of complications and a postoperative hysterosalpingogram (3 months after the surgery) demonstrated no uterine parietal defect nether uterine diverticulitis. The patient had become spontaneously pregnant 11 months after the surgery and is actually at 32 weeks of gestation.

## 3. Discussion

The pathological definition of an intramyometrial pregnancy refers to a conceptus implanting within the myometrium and separated from both the uterine cavity and tubes as well as surrounded by myometrium. It is amongst the rarest type of ectopic pregnancy and constitutes less than 1% of their total number. This type of pregnancy very rarely goes further than 12 weeks' gestation, where the risk of uterine rupture is increased and the percentage of maternal mortality is approximately 2.5% [1]

### 3.1. Pathophysiology

There are many theories for the etiological factors of this rare ectopic pregnancy. The most commonly cited aetiological factors is previous uterine trauma resulting in a sinus tract within the endometrium. Other aetiological factors are increased trophoblastic activity and defective decidualisation which allow the conceptus to penetrate into the myometrium. Implantation on the focus of intramural adenomyosis may also account for this phenomenon as may serosal implantation of the conceptus following external migration. It is also known to be associated with in vitro fertilisation and embryo transfer. Traumatic factors, such as dilatation and curettage (D&C), cesarean section, myomectomy, manual removal of the placenta, or even difficult embryo transfer, are implicated in most cases [2].

Intramural pregnancy after embryo transfer has been reported in the literature, where the creation of a false passage at a previous instrumentation of the cervix may be implicated in the ectopic placement of embryos [2].

### 3.2. Diagnosis

Pelvic pain and uterine bleeding in the presence of a positive pregnancy test are the hallmark presentation of ectopic pregnancy. However, early diagnosis of intramyometrial pregnancy is very difficult and always made intraoperatively. Only three cases of intramural pregnancy have been correctly diagnosed preoperatively by ultrasound and one by magnetic resonance imaging [3, 4].

The typical ultrasound appearance of intramural pregnancy is a gestational sac completely surrounded by myometrium. The ultrasound appearance can mimic degenerating fibroid, congenital uterine anomaly, or pregnancy in a sacculation or diverticulum. Some authors reported the use of hysteroscopy which allows for direct observation of the uterine cavity and tubal ostium and confirms the absence of the conceptus in the uterine cavity. Serial *β*-hCG assay has been reported to be useful for the diagnosis [3].

### 3.3. Treatment

The majority of reported cases of intramyometrial pregnancy were managed by hysterectomy [3]. This high rate of hysterectomy probably reflects delayed diagnosis. Laparotomy with hysterectomy was the treatment of choice in most cases until 5 years ago when transvaginal sonography allowed the early diagnosis and conservative treatment of the condition in infertile patients [5].

There are few cases of conservative management with surgical excision. In our case even with delayed diagnosis and the rupture of uterine wall a conservative management was successfully performed.

Some authors reported a successful medical treatment with local injection of methotrexate avoiding surgery [2, 4, 5].

## 4. Conclusion

Even intramyometrial pregnancy is a very rare type of ectopic pregnancy it should be kept in mind by gynecologist because it can become a life-threatening condition. Early diagnosis is therefore very important, since it makes conservative treatment possible and helps preserve fertility.
